# Resistant dextrin reduces obesity and attenuates adipose tissue inflammation in high-fat diet-fed mice

**DOI:** 10.7150/ijms.45723

**Published:** 2020-09-20

**Authors:** Qiuyue Hu, Yao Lu, Fan Hu, Sunyue He, Xiaoyuan Xu, Yixin Niu, Hongmei Zhang, Xiaoyong Li, Qing Su

**Affiliations:** Department of Endocrinology, Xinhua Hospital, Shanghai JiaoTong University School of Medicine, 1665 Kongjiang Road, Shanghai 200092, China.

**Keywords:** Resistant dextrin, Obesity, Adipose tissue inflammation, NF-κB signaling pathway

## Abstract

Resistant dextrin (RD), a short chain glucose polymer, has been shown to improve type 2 diabetes mellitus (T2DM) in clinical studies. However, the improvement of adipose tissue inflammation and specific mechanisms of RD supplementation in obesity have not been fully investigated. Therefore, we examined whether RD attenuates obesity and adipose tissue inflammation in high-fat diet (HFD)-fed mice. Male C57BL/6 mice were fed a chow diet, a HFD or a HFD with RD supplementation for 12 weeks. Body weight (BW), fasting blood glucose (FBG), epididymal fat accumulation, serum total triglyceride (TG), free fatty acid (FFA) and inflammatory cytokine levels (TNF-α, IL-1β, IL-6, IL-10) were measured. Inflammation markers and macrophage infiltration in epididymal adipose tissue were observed. After 12 weeks of intervention, the body weight gain of mice in RD supplementation group was less than that in HFD group. FBG, epididymal fat accumulation, serum TG and FFA levels were reduced in RD supplementation group compared with HFD group. Moreover, serum and mRNA levels of IL-6 were significantly reduced in the RD supplementation group. In addition, RD supplementation reduced macrophage infiltration, regulated polarization of macrophage and inhibited NF-κB signaling in epididymal adipose tissue. In conclusion, RD reduces obesity and attenuates adipose tissue inflammation in HFD-fed mice, and the inhibition of NF-κB signaling may be a presumed mechanism for its effects.

## Introduction

Obesity, a worldwide epidemic, has contributed to a series of health problems for both individuals and society 1. Obesity involves the excess accumulation of body fat and may contribute to multiple metabolic diseases, including type 2 diabetes, cardiovascular disease, fatty liver disease, cancers and stroke 1-5. In addition, obesity increases the risk of joint pain, arthritis, sleep apnea and mental disorders 2, 6. Therefore, effective treatment measures are urgently needed for this global health problem. Diet, exercise, anti-obesity drugs and bariatric surgery are the main approaches to prevent and treat obesity 7. However, lifestyle modifications and drugs are often not largely effective in preventing obesity and maintaining weight loss over time 7, 8. Although bariatric surgery is largely beneficial to weight loss and reduces adverse health consequences 5, 8-10, long-term health outcomes of bariatric surgery are regrettably unclear and it is not accessible to people in low- and middle-income countries 11. Thus, a novel intervention strategy is crucial to solve or alleviate this major global problem.

Dietary fiber, defined as carbohydrate polymers that are neither digested nor absorbed in the human small intestine, is obtained from food raw materials and has physiological benefits 12. Previous studies have demonstrated that dietary fiber is negatively associated with the risk of dyslipidemia, hypertension, obesity, diabetes, peripheral vascular disease, coronary heart disease and stroke 13. Resistant dextrin (RD) is a soluble dietary fiber and prebiotic, which has been widely used in functional food and beverage products due to its low viscosity and great water solubility 12, 14, 15. RD is rich in α-1,4 and α-1,6 linkages and derives from wheat or maize 14, 16. In addition, RD partially resists enzymatic hydrolysis in the human gastrointestinal tract 14, 17: approximately 15% of RD is digested in the small intestine, 75% is fermented in the colon, and approximately 10% is excreted in the feces 18. Short-term and long-term gastrointestinal tolerance studies of RD have shown that the fiber supplementation is well tolerated up to a dose of 45 g daily in healthy men 19, 20.

Moreover, RD supplementation was shown to regulate metabolic parameters and androgen levels in women with polycystic ovary syndrome (PCOS) 18. RD supplementation has also been shown to improve magnesium and calcium absorption and retention 21. Although a previous study has shown that body weight is reduced in overweight men who are given 17 g RD twice daily 22, the improvement of adipose tissue inflammation and specific mechanisms of RD supplementation in HFD-fed induced obese mice are not still investigated.

In the present study, our results show that RD reduces obesity and attenuates adipose tissue inflammation in HFD-fed mice. Moreover, RD supplementation reduces macrophage infiltration, regulates polarization of macrophage and inhibits the NF-κB signaling pathway in epididymal adipose tissue. Our study demonstrates that RD may represent a new non-drug therapy to prevent and treat obesity.

## Materials and Methods

### Animals and sample collection

Male C57BL/6 mice, aged four weeks, were purchased from the Shanghai SLAC Laboratory Animal Co. Ltd. Four animals were housed per cage with free access to food and sterile drinking water in a climate-controlled room (21°C ± 2°C) under a 12-hour light/dark cycle (7:00 AM-7:00 PM). After a week of acclimatization, mice were randomly divided into three groups: control chow group (chow), high-fat diet group (HFD) and high-fat diet supplemented with resistant dextrin group (HFD+RD), and each group consisted of 12 mice. The chow group mice were fed for 12 weeks with a standard diet, and the other two groups were fed with a HFD (45% energy from fat; D12451, Research Diet, USA). In addition, the HFD+RD group mice were orally administered 10 g/kg RD, and the other two groups were orally administered equal sterile saline once per day for 12 weeks. All procedures were performed in accordance with the National Institutes of Health Guidelines for the Care and Use of Animals and were approved by the Ethics Committee of Xinhua Hospital affiliated with Shanghai Jiao Tong University School of Medicine.

Mice were fasted overnight for 16 h then anesthetized, and whole blood samples were collected via eyeball extraction. Epididymal white adipose tissue was sampled and weighed. Tissue samples were immediately placed in liquid nitrogen and stored at -80°C before the assay.

### Reagents and antibodies

RD was purchased from Guangzhou Honsea Industry Co. Ltd. Rabbit monoclonal antibodies against phospho-Ser32 IκBα (#2859), p65 (#8242), phospho-Ser536 p65 (#3033), β-actin (#4970) and a mouse monoclonal antibody against IκBα (#4814) were purchased from Cell Signaling Technology (Cell Signaling, USA). Goat polyclonal secondary antibodies against mouse (#A0216) or rabbit (#A0208) were purchased from Beyotime Biotechnology (Beyotime, China).

### Histological analysis and immunohistochemical staining

Epididymal white adipose tissue samples from all groups of mice were fixed in phosphate-buffered 10% formalin and embedded in paraffin wax. On one hand, sections were cut (4 μm thick) and stained with hematoxylin and eosin (H&E). The quantification of adipocyte size was performed in five microscopy fields for each mouse using ImageJ software (magnification 200×). On the other hand, the slices were dewaxed with xylene and rehydrated with graded ethanol to water. The slices were then placed in citric acid antigen repair buffer and heated in a microwave oven for antigen repair. Slices were incubated with 3% H_2_O_2_ in the dark at room temperature for 25 minutes to block the activity of endogenous peroxidase. 3% BSA was used for background blocking for 30 minutes. The slices were incubated with primary rabbit anti-mouse F4/80 at 4°C overnight. After washing the slices three times, slices were incubated with HRP-conjugated secondary antibody for 50 min. After washing the slices another three times, color was developed using the diaminobenzidine substrate and counterstained with hematoxylin. Microscope was used to collect pictures, then ImageJ was used to calculate the percentage of F4/80-positive staining area per slice 23.

### Serum total triglyceride measurements

Blood samples were centrifuged, and plasma was collected and stored at -80°C before the assay. Total triglyceride (TG) levels of all the samples were tested in an automatic biochemical analyzer (Hitachi, Japan).

### Serum free fatty acid measurements

Serum free fatty acid (FFA) levels were measured using commercial kits (Nanjing Jiancheng Bioengineering Institute, China).

### Enzyme-linked immunosorbent assay (ELISA)

Serum lipopolysaccharide (LPS), tumor necrosis factor-alpha (TNF-α), interleukin-1β (IL-1β), interleukin-6 (IL-6) and interleukin-10 (IL-10) levels were quantified according to the protocol of the corresponding ELISA kit (Westang, China).

### Quantitative real-time polymerase chain reaction (PCR)

Total RNA was extracted from frozen tissues using Trizol reagent according to the manufacturer's instructions (Takara, Japan). Then, the total RNA was converted to complementary deoxyribonucleic acid (cDNA) using a reverse transcription reagent kit (Takara, Japan). Quantitative real-time PCR was performed with SYBR Green PCR reagent (Takara, Japan) for quantification of the mRNA levels. Calculations were made based on the comparative cycle threshold method (2^-ΔΔCt^). The chow group was used as calibrator group and glyceraldehyde 3-phosphate dehydrogenase (GAPDH) was used as an endogenous normalization control. Primer sequences are given in Table 1.

### Western blot analysis

RIPA lysis buffer containing protease and phosphatase inhibitor cocktails was used to extract proteins. Then, the extracted proteins were quantified with a BCA Protein Quantitative Analysis kit (Beyotime, China). A total of 20 μg of the protein sample was separated on an SDS PAGE gel and transferred to a polyvinylidene fluoride (PVDF) membrane (Millipore, USA). After that, the membrane was blocked with 5% skim milk powder and incubated overnight with primary antibodies. Next, the membrane was washed three times and incubated with an HRP-conjugated secondary antibody. Finally, the membrane was washed another three times, and the protein bands were detected with Immobilon Western Chemiluminescent HRP Substrate (Millipore, USA). ImageJ software was used to quantify the protein bands.

### Statistical analysis

All data are shown as the mean ± standard error of the mean (SEM). All statistical analyses were performed using GraphPad Prism V.7.0. One-way or two-way analysis of variance (ANOVA) followed by Bonferroni's post hoc test was used for comparison of multiple groups. A two-tailed unpaired t test was used for comparison of two groups. A value of *p* < 0.05 was considered statistically significant.

## Results

### RD reduced HFD-induced obesity in mice

**Figure 1:**
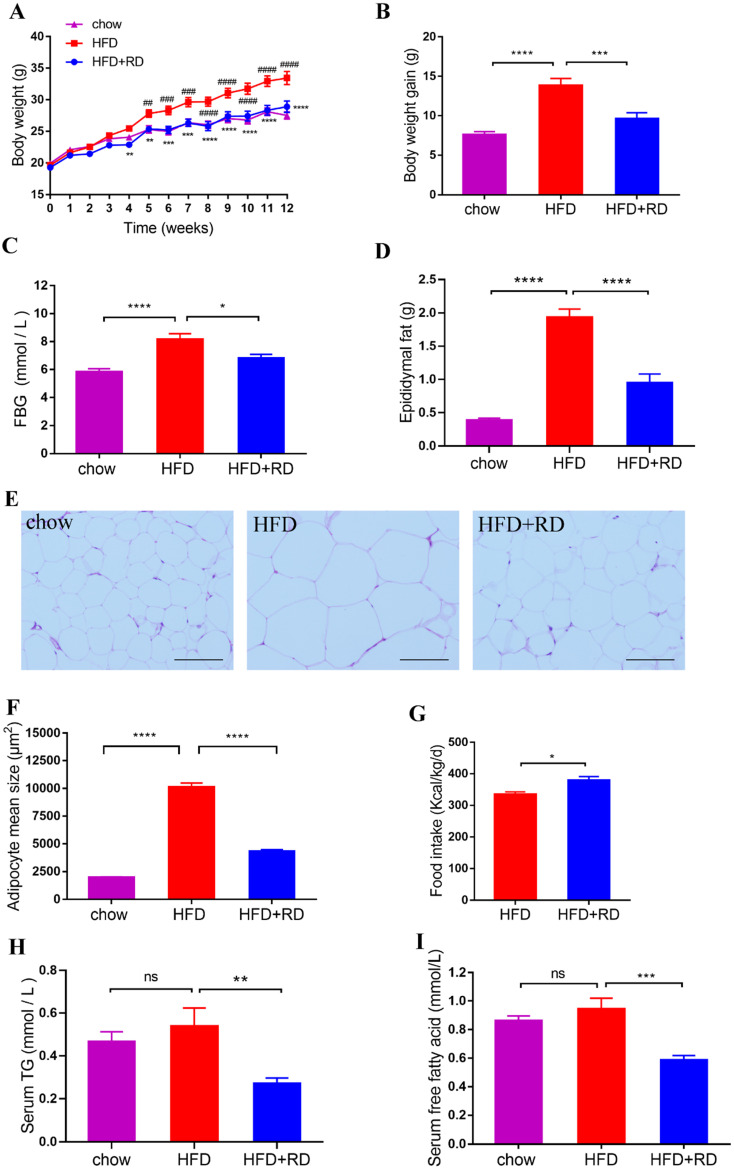
** RD reduces HFD-induced obesity in mice.** Chow- and HFD-fed mice were treated daily with sterile saline or RD by intragastric gavage for 12 weeks. (A) Body weight. (B) Body weight gain at week 12. (C) FBG at week 12. (D) Epididymal fat accumulation. (E) Representative images of hematoxylin and eosin (H&E)-stained epididymal adipose tissues. Scale bar, 100 µm; magnification, ✕200. (F) Adipocyte size in epididymal adipose tissues was determined using Image J software. (G) Food intake at week 12. (H) Serum TG. (I) Serum free fatty acid. Data are presented as mean ± SEM, n=12 mice per group**.** Statistical analysis was performed using two-way (panel A) or one-way (Except panels A and G) analysis of variance (ANOVA) followed by Bonferroni's post hoc test; Panel G was analyzed by unpaired t test. ^*^p<0.05; ^**^p<0.01; ^***^p<0.001; ^****^p<0.0001; ns, not statistically significant. In panel A**,** ^**^p<0.01; ^***^p<0.001; ^****^p<0.0001 versus HFD; ^##^*p<*0.01; ^###^*p<*0.001;^ ####^*p<*0.0001 versus chow.

To study the effects of RD on obesity, the obesity model induced by HFD was used in this study. After 12 weeks of HFD feeding, the BW, body weight gain, FBG, epididymal fat accumulation and adipocyte size were increased significantly compared with control chow group (Fig. 1A-F). However, RD treatment reduced the BW, body weight gain, FBG, epididymal fat accumulation and adipocyte size compared with HFD group (Fig. 1A-F). In addition, RD supplementation increased food intake compared with HFD group (Fig. 1G). Adipose tissue can store nutrients in the form of triglycerides. To investigate whether RD alleviates the serum lipid level, a quantitative analysis of serum TG and FFA levels was performed. We observed that RD supplementation reduced serum TG and FFA levels in HFD-fed mice (Fig. 1H & I). These results indicate that RD prevents HFD-induced obesity in mice.

### RD reduces proinflammatory cytokine levels in HFD-fed mice

**Figure 2:**
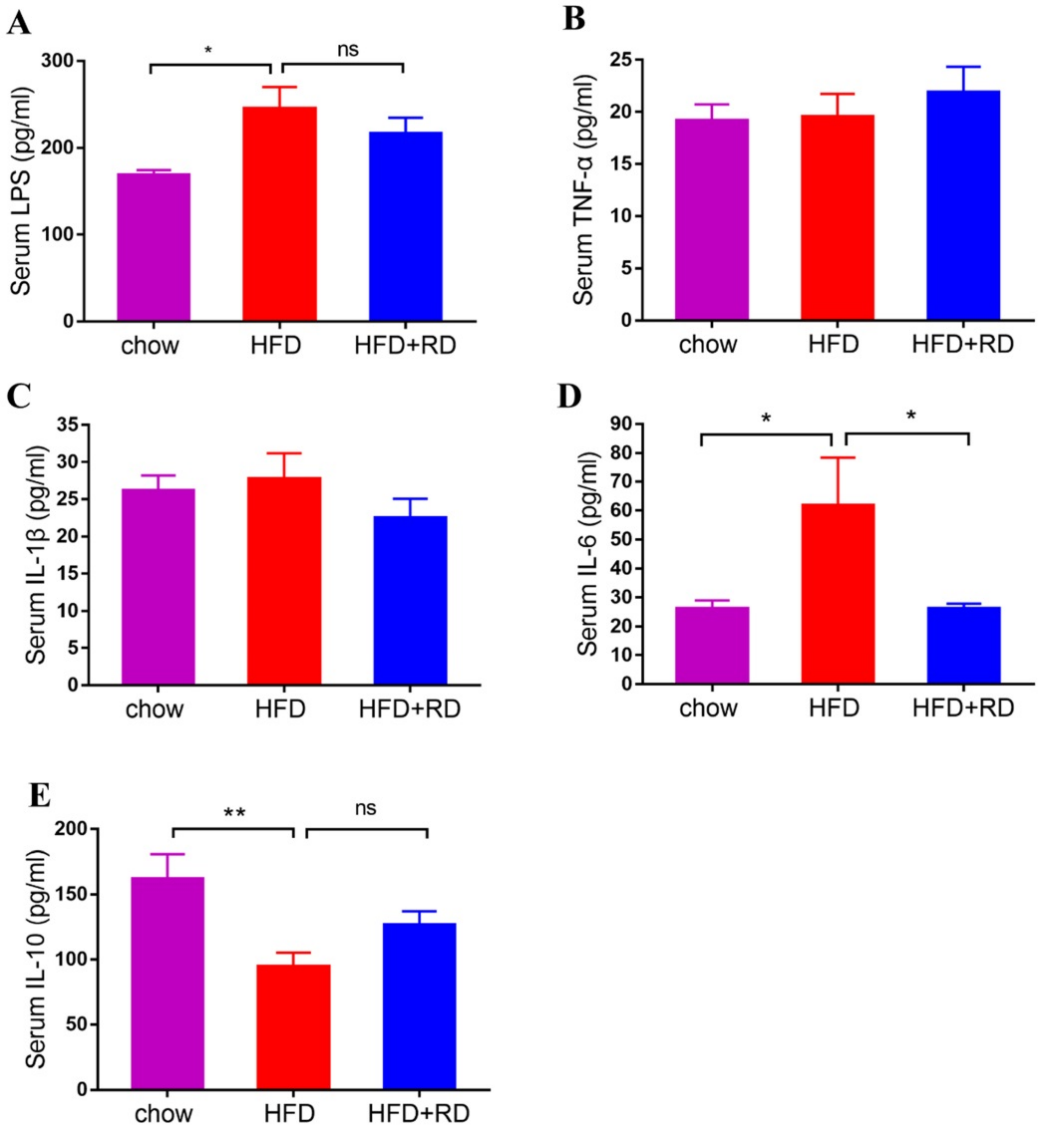
** RD reduces proinflammatory cytokine levels in HFD-fed mice.** (A-E) Serum LPS (A), TNF-α (B), IL-1β (C), IL-6 (D), IL-10 (E) levels were determined using ELISA at week 12. N=12 mice per group. Data are presented as mean ± SEM. Statistical analysis was performed using one-way analysis of variance (ANOVA) followed by Bonferroni's post hoc test. ^*^p<0.05; ^**^p<0.01; ^***^p<0.001; ^****^p<0.0001; ns, not statistically significant.

**Figure 3:**
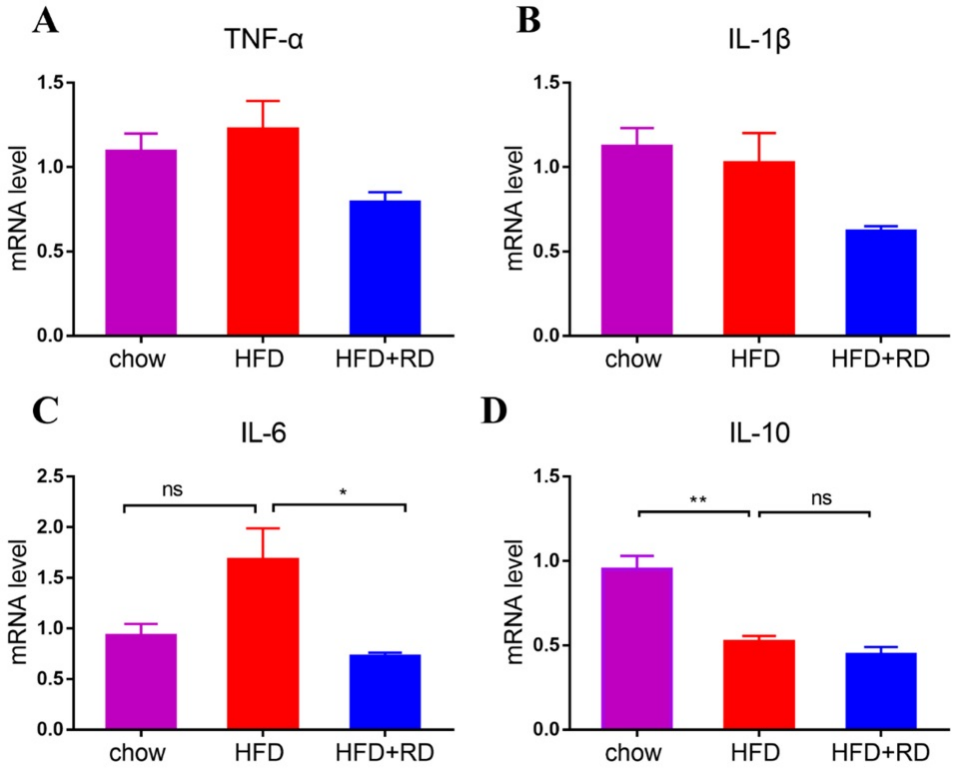
** RD reduces the expression levels of proinflammatory genes in HFD-fed mice.** (A-C) qPCR analysis of proinflammatory related genes TNF-α (A), IL-1β (B), and IL-6 (C) levels in epididymal adipose tissue. N = 4 mice per group. (D) qPCR analysis of anti-inflammatory gene IL-10 level in epididymal adipose tissue. N = 4 mice per group. Data are presented as mean ± SEM. Statistical analysis was performed using one-way analysis of variance (ANOVA) followed by Bonferroni's post hoc test. ^*^p<0.05; ^**^p<0.01; ^***^p<0.001; ^****^p<0.0001; ns, not statistically significant.

Obesity-associated chronic tissue inflammation is a key contributory factor to type 2 diabetes and cardiovascular disease, and previous studies have clearly suggested that metabolism and immune system are closely integrated 24. To determine whether RD regulates inflammatory cytokine levels, we performed a quantitative analysis of the serum LPS, TNF-α, IL-1β, IL-6, and IL-10 levels. We observed that LPS and IL-6 levels were higher in HFD-fed mice compared with chow-fed mice, and serum IL-6 level was significantly reduced in the RD supplementation group compared with the HFD group (Fig. 2A & D). Moreover, HFD reduced the serum level of the anti-inflammatory cytokine IL-10, which was slightly increased in RD supplementation group (Fig. 2E). Next, we examined the mRNA expression levels of proinflammatory genes and anti-inflammatory genes, including TNF-α, IL-1β, IL-6, and IL-10, in epididymal adipose tissue. Similar to the serum inflammatory cytokine result, the mRNA level of IL-6 was reduced by RD supplementation (Fig. 3C). However, no significant alteration was found in the mRNA expression levels of TNF-α, IL-1β, and IL-10 (Fig. 3A, B & D). These results indicate that RD supplementation reduces proinflammatory cytokine expression and production in HFD-fed mice.

### RD supplementation reduces macrophage infiltration in epididymal adipose tissue of HFD-fed mice

**Figures 4:**
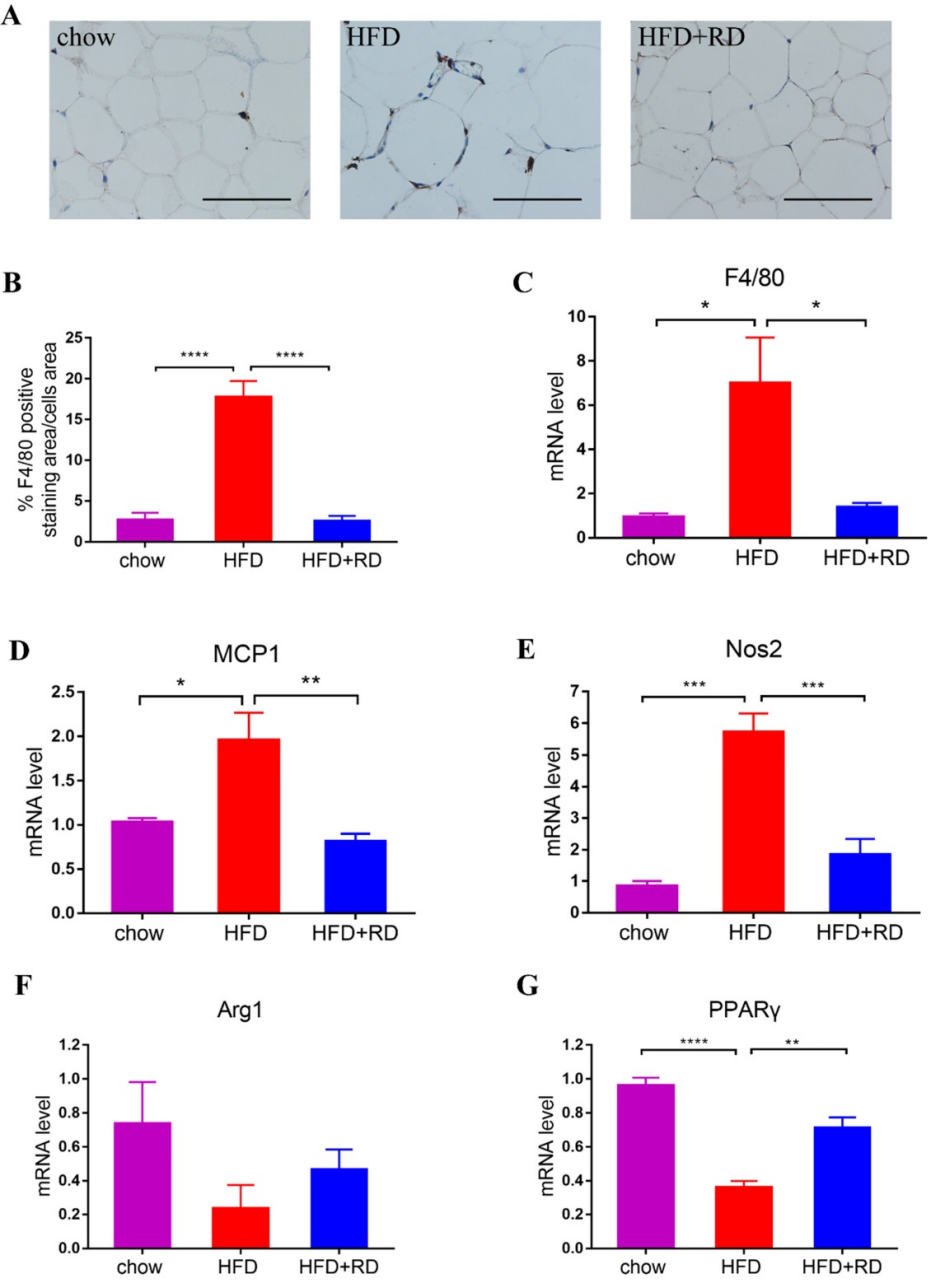
** RD supplementation reduces macrophage infiltration in epididymal adipose tissue of HFD-fed mice.** (A) Representative images of immunohistochemical stain of epididymal adipose tissue against the specific macrophage maker F4/80. Scale bar, 100 µm; magnification, ✕200. (B) Quantification of F4/80-positive staining area in epididymal adipose tissue. N=6 mice per group. (C-G) qPCR analysis of the mRNA level of F4/80 (C), MCP1 (D), Nos2 (E), Arg1 (F) and PPARγ (G) in epididymal adipose tissue. N=4 mice per group. Data are presented as mean ± SEM. Statistical analysis was performed using one-way analysis of variance (ANOVA) followed by Bonferroni's post hoc test. ^*^p<0.05; ^**^p<0.01; ^***^p<0.001; ^****^p<0.0001; ns, not statistically significant.

Obesity is characterized by infiltration and activation of macrophages in adipose tissues, which results chronic low-grade inflammation 25. Therefore, the quantification of F4/80-positive macrophages was observed by immunohistochemical staining in epididymal white adipose tissue. The results showed that the percentage of F4/80-positive staining area was increased in HFD group compared with chow group and RD supplementation significantly reversed macrophage infiltration (Fig. 4A & B). In addition, the mRNA level of F4/80 was also significantly reduced in RD supplementation group compared with HFD-fed mice (Fig. 4C). The mRNA levels of proinflammatory cytokines monocyte chemoattractant protein 1 (MCP1) and M1 macrophage marker nitric oxide synthase 2 (Nos2) were all increased in HFD-fed mice compared with chow-fed mice. RD supplementation significantly reversed MCP1 and Nos2 gene expression levels in HFD-fed mice (Fig. 4D-E). Moreover, the mRNA level of M2 macrophage marker peroxisome proliferators-activated receptor γ (PPARγ) was reduced in HFD-fed mice compared with chow group. RD supplementation significantly increased PPARγ expression level and also tended to increase Arg1 level compared with HFD group (Fig. 4F-G). These results indicate that RD supplementation reduces macrophage infiltration and regulates polarization of macrophage in epididymal adipose tissue of HFD-fed mice.

### RD inhibits NF-κB signaling pathways in the adipose tissue of HFD-fed mice

**Figure 5:**
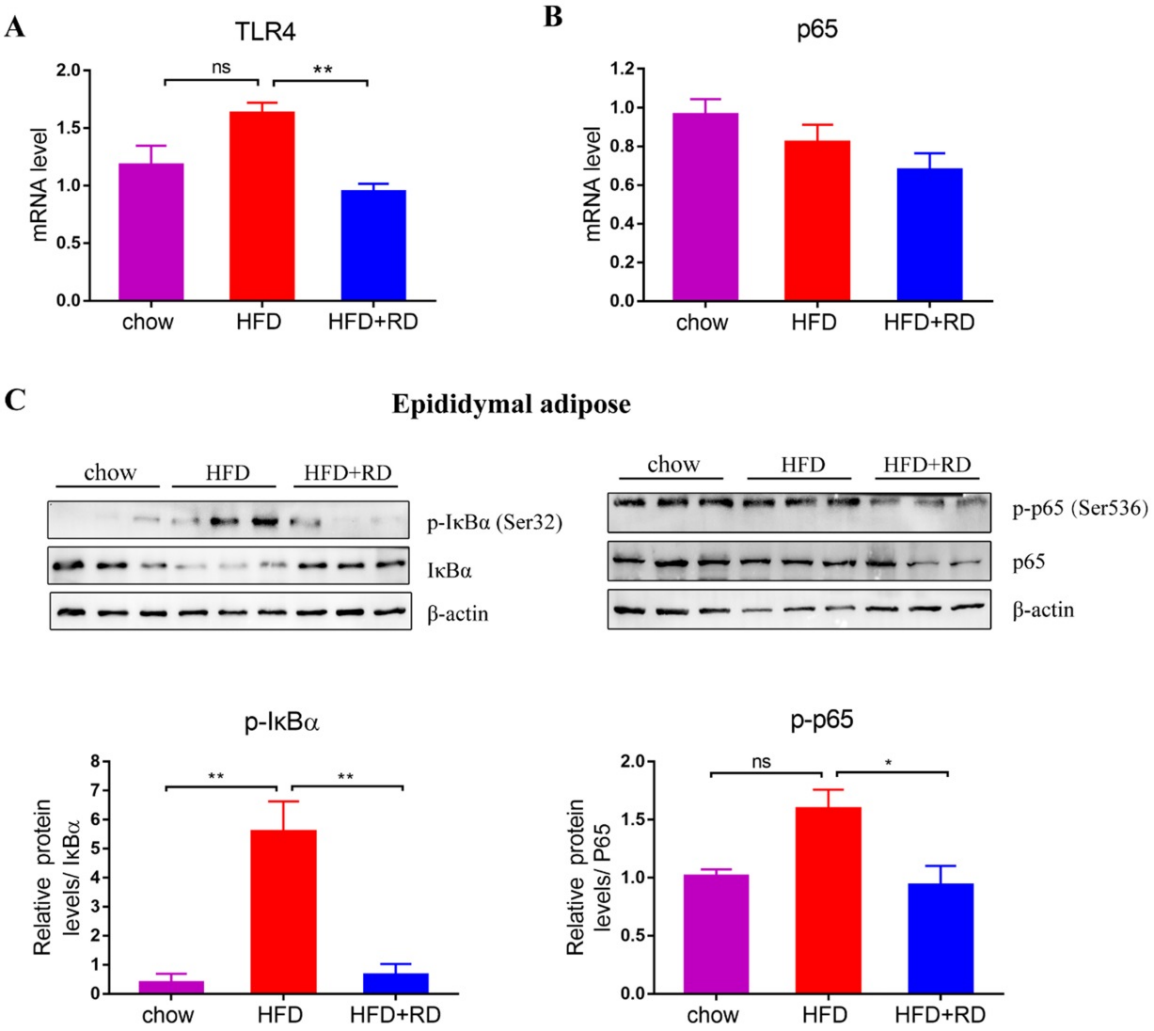
** RD inhibits NF-κB signaling pathways in epididymal adipose tissue of HFD-fed mice.** (A-B) qPCR analysis of the mRNA level of TLR4 (A) and p65 (B) in epididymal adipose tissue. N=4 mice per group. (C) Western blot analysis of NF-κB signaling pathway related proteins expression levels in epididymal adipose tissue. N=3 mice per group. Data are presented as mean ± SEM. Statistical analysis was performed using one-way analysis of variance (ANOVA) followed by Bonferroni's post hoc test. ^*^p<0.05; ^**^p<0.01; ^***^p<0.001; ^****^p<0.0001; ns, not statistically significant.

Previous studies have indicated that FFA and LPS might promote adipose tissue inflammation by binding to Toll-like receptors (TLRs), such as TLR4 26. TLR4 activates downstream NF‑κB signaling to increases the synthesis and secretion of MCP1, which promotes macrophage recruitment to adipose tissue and the development of insulin resistance 27-29. Therefore, we further explored the mRNA expression level of TLR4. The results showed that the mRNA level of TLR4 in the epididymal white adipose tissue of RD-treated mice was lower than that of HFD-fed mice (Fig. 5A). Moreover, we examined whether the NF‑κB signaling pathway was affected by RD supplementation in epididymal white adipose tissue. The protein expression level of IκBα, which is an inhibitor of the transcription factor NF-κB, was upregulated in RD-treated mice compared with HFD-fed mice. In contrast, the level of phospho-Ser32 IκBα, which leads to the release of active NF-κB and nuclear translocation, was reduced in RD-supplemented mice compared with HFD-fed mice (Fig. 5C). RD supplementation reduced phospho-Ser536 p65 expression level compared with HFD group (Fig. 5C). These results indicate that RD supplementation inhibits NF-κB signaling in epididymal adipose tissue of HFD-fed mice.

## Discussion

Several randomized clinical trials have shown that RD improves **i**nsulin resistance, glycemic control, and blood pressure and modulates inflammation 16, 17, 30, 31. A previous study shown that body weight was reduced in overweight men who were given 17 g RD supplementation twice daily 22. However, the improvement of adipose tissue inflammation and specific mechanism of RD in HFD-induced obesity were not fully understood. In the present study, we mainly studied the effects of RD on epididymal fat, which is the typical representative of visceral fat and closely associated with obesity-related diseases. We found that RD reduced obesity and attenuated adipose tissue inflammation in HFD-fed mice, including reducing body weight gain, FBG, epididymal fat accumulation, adipocyte size, proinflammatory cytokines and macrophage infiltration. Our research suggests that RD may represent a new non-drug therapy to prevent and treat obesity.

Many studies have indicated that HFD can induce adipocyte expansion in size and number, which aims to accommodate the need for excessive TG storage and the anabolic force of hyperinsulinemia 32-34. Our study shown that RD reduced visceral fat accumulation, adipocyte hypertrophy and serum TG. When adipocytes reach a certain metabolic stress, an inflammatory program is to be triggered. The triggers of the inflammatory program may originate from a gut-derived substance, dietary component and metabolite 26. Previous studies have shown that HFD can disrupt gut barrier integrity and lead to translocation of LPS from gram-negative bacteria into the blood, which produces inflammation and insulin resistance 35, 36. Moreover, elevated concentration of free fatty acid in HFD-fed mice can also contribute to adipose tissue inflammation. High circulating levels of LPS and FFA can cause systemic and targeted inflammation in HFD-fed mice by activating TLR4 signaling 37-39. A previous study has shown that TLR4^-/-^ HFD-fed mice significantly reduces adipose tissue inflammatory markers and macrophage recruitment 40. We found that RD reduced the serum levels of FFA and LPS, and the mRNA level of TLR4 was also reduced in epididymal adipose tissue.

HFD activates several proinflammatory signaling pathways, and NF-κB signaling is a crucial pathway in this process, which is the downstream of TLR4 and leads to the development of insulin resistance, cytokine production, and eventually immune cell recruitment 41. A previous study has shown that inhibition of inflammatory signaling by knockout of NF‑κB pathway can disrupt the link between obesity and insulin resistance in obese mice 42. Another study shown that stevioside (SVS), a widely used sweetener with multiple beneficial effects for diabetic patients, reduced expression levels of TNF-α, IL-6, IL-1β, macrophage inflammatory protein-1α (MIP-1α), CD11b and CD14 and inhibited the NF-κB signaling pathway in adipose tissue 43. Moreover, a water extract of Ganoderma lucidum mycelium (WEGL) reduced endotoxemia and enhanced the expression of IκBα in adipose tissue in HFD mice 32. In the present study, we found that RD increased the protein expression level of IκBα, which interacts with the transcription factor NF-κB and prevents NF-κB translocation and activation. In addition, the protein level of p-IκBα was reduced in RD-supplemented mice compared with HFD-fed mice. Moreover, RD supplementation reduced the p-p65 expression levels compared with HFD group. Activation of NF‑κB signaling pathway can increase the synthesis and secretion of MCP1 in adipocytes. MCP1 can promote macrophage recruitment to adipose tissue and the development of insulin resistance. Our results showed that the mRNA level of MCP1 was upregulated significantly in the HFD-fed mice, which was reversed by RD supplementation.

Many studies have demonstrated that obesity is closely associated with an overall increase of macrophage infiltration in both rodents and humans 44. Macrophages are generally grouped as M1 and M2 phenotypes, which respectively represent pro- or anti-inflammatory phenotypes. The expression of maker genes for M1 and M2 macrophages from mouse epididymal fat tissue are different. TNF-α, IL-1β, IL-6 and Nos2 were highly expressed in the M1 macrophages, and CD206, CD163, IL-10, Arg1, PPARγ, Mrc1 and Fizz1 were highly expressed in the M2 macrophages 45, 46. The macrophages that appear in large numbers in the adipose tissue of obese people are often referred to as M1 macrophages 47. On the one hand, pro‑inflammatory macrophages are recruited from blood monocytes to adipose tissue 48, on the other hand, resident classically activated macrophages surround the dead adipocytes and form crown‑like structures (CLS) 49. In the present study, we found that RD reduced macrophage infiltration and regulated polarization of macrophage towards the M2 type in epididymal adipose tissue of HFD-fed mice, which reduced IL-6 and Nos 2 expression levels and increased PPARγ level.

The gut microbiota of obese human and mice is associated with decreased level of intestinal Bacteroidetes and increased level of Firmicutes 50, 51. Many studies have been performed to investigate the effect of dietary fiber on the modulation of gut microbiota. Dietary fibers can alleviate type 2 diabetes by promoting a specific group of short-chain fatty acid (SCFA)-producing gut bacteria 52. Inulin supplementation alleviates glucose and lipid metabolism disorders by modulating the gut microbiota in ob/ob mice 53. The major metabolites from the microbial fermentative activity in the gut are SCFAs, including acetate, propionate, and butyrate. GPR41, GPR43 and GPR109A are considered SCFAs receptors 54, 55. A study showed that the GPR109A agonist butyrate reduced LPS-induced NF-κB activation in mouse colons and KM12L4 cancer cell line 56. GPR43 plays an important role in white adipose tissue. Adipose-specific overexpression of GPR43 mice can remain lean, even when the mice were fed a HFD. However, the effects were abrogated when mice were raised under germ-free conditions 57. Moreover, a previous study has indicated that RD from maize starch induces the growth of Bacteroidetes and Actinobacteria and inhibits the growth of Firmicutes in obesity 58. Another study has shown that RD contributes to the growth of butyrate- and acetate-producing microbes in male IL-10^-/-^mice 59. Therefore, the modulation of gut microbiota may also play an important role in metabolically beneficial effects of RD supplementation mice.

## Conclusions

In summary, RD supplementation reduces body weight gain, FBG, visceral fat accumulation, adipocyte hypertrophy, proinflammatory cytokines, macrophages infiltration and regulates polarization of macrophage in epididymal adipose tissue of HFD-fed mice. Moreover, RD supplementation inhibits NF-κB signaling in epididymal adipose tissue, which may be a presumed mechanism for metabolically beneficial effects. Our research suggests that RD may represent a new non-drug therapy to prevent and treat the major global problem obesity.

## Figures and Tables

**Table 1:** Sequence of primers for quantitative real-time PCR

Gene	Species	Forward primer	Reverse primer
TNF-α	Mouse	ATGTCTCAGCCTCTTCTCATTC	GCTTGTCACTCGAATTTTGAGA
IL-1β	Mouse	TCGCAGCAGCACATCAACAAGAG	AGGTCCACGGGAAAGACACAGG
IL-6	Mouse	CTGCAAGAGACTTCCATCCAG	AGTGGTATAGACAGGTCTGTTGG
IL-10	Mouse	TTCTTTCAAACAAAGGACCAGC	GCAACCCAAGTAACCCTTAAAG
F4/80	Mouse	TGTCTGCATGATCATCACGATA	CGTGTCCTTGAGTTTAGAGACT
MCP1	Mouse	TTTTTGTCACCAAGCTCAAGAG	TTCTGATCTCATTTGGTTCCGA
Nos2	Mouse	AGGCCACATCGGATTTCACT	TCAATGGCATGAGGCAGGAG
TLR4	Mouse	GCCATCATTATGAGTGCCAATT	AGGGATAAGAACGCTGAGAATT
Arg1	Mouse	AGACCACAGTCTGGCAGTTGG	AGGTTGCCCATGCAGATTCCC
PPAR γ	Mouse	CCAAGAATACCAAAGTGCGATC	TCACAAGCATGAACTCCATAGT
P65	Mouse	ATTTCCGCCTCTGGCGAATG	GATGAGGGGAAACAGATCGTCC
GAPDH	Mouse	AAGAAGGTGGTGAAGCAGGCATC	CGGCATCGAAGGTGGAAGAGTG
